# Supplementary material for: Unveiling Species Diversity Within Early-Diverging Fungi from China VIII: Four New Species in Mortierellaceae (Mortierellomycota)
Source: Microorganisms. 2025 Jun 7;13(6):1330. doi: 10.3390/microorganisms13061330 (PMC12195542; doi:10.3390/microorganisms13061330)
Supplement: Supplementary file 1 [file microorganisms-13-01330-s001.zip › Morphological table.pdf]

Table S3. On the morphological characterization of species in the genus *Mortierella and Linnemannia*.

Species	Colonies	Sporangiophores	Sporangia	Chlamydospores	Sporangiospores	Reference
Mortierella acuta	PDA:16°C 6d, 64mm, garlic smell, white cottony, with a rosette pattern.	Erect or slightly bent, unbranched, 22.4–72.4 μm in height, tapering from 2.8–4.3 μm to at the base to 1.0–1.4 μm at the apex.	Almost spherical in shape, smooth, deliquescent, 9.5–12.6 μm in diameter.	Absent	Transparent, mostly oval, 2.6–3.4 μm long, 1.4–1.7 μm wide.	This study
M. alpina	Colonies zonate or lobate; aerial mycelia abundant, white, cottony; colonies 30-60 mm after 5 d at 20°C on MA: garlic-like odour strong or weak.	Sporangiophores arising from aerial and substrate mycelia, always unbranched, swollen at the bases; 20-140(-190) um long, tapering from (2.5-3.0=6.0(-8.0) um wide at the base to (0.5-)1.0-3.0 um at the tip.	Many-spored: irregular, globose or subglobose, 8–22 μm diam. Single-spored: Ellipsoidal: 4–10 × 10–17μm. Globose: 6–18μm diam.	Chlamydospores absent to abundant, ellipsoidal, lemon-shaped or subglobose, (4-)6-12(-20) um diam.	Sporangiospores cylindrical, ellipsoidal or subglobose, (1.0-)1.5-2.5(-4.0) × (1.0-) 2.5-4.0(-7.0) um.	[44]
M. biramosa	A radiate pattern with cottony aerial mycelia on PDA and V8A. PDA, V8A, and CMA: >70 mm at 25°C, after 3d.	Sporangiophores 300-1,500 μm long.	Sporangia globose; 30-40 μm in diameter.	Chlamydospores globose; 15-35 μm in diameter.	Sporangiospores globose; smooth-walled with granular contents 5-8 μm in diameter.	[50]
M. calciphila	Radiate colonies fast-growing (6–9 mm per day on PDA), without characteristic zonate growth nor garlic odour.	Sporangiophores: arising from substratum (with 2–4 basal sympodial ramifications) or aerial hyphae (with 0–1 ramification); slender, 2–3 μm under sporangium, 600–1400 μm long, aseptate.	Sporangia (27–) 70 (–80) μm in diam., many-spored, with early deliquescent wall.	Absent	Spores broadly ellipsoidal, smooth-walled, regular in shape (8–)9(–11) μm (SD = 0.8) × (6–)7(–9) μm (SD = 0.8).	[51]

M. dichotoma	Not described	sporangiophores 200-750 um long.	Sporangia 20-40 um diam.	Chlamydospores 5-10 um diam.	Sporangiospores 4-7 × 2.5-4 um.	[4]
M. gemmifera	White, inconspicuously densely lobate on MA; abundant aerial mycelia; weak indistinct odour; 40 mm after 5 d at 20°C; sparse aerial mycelia on PCA and SEA.	Sporangiophores: arising from aerial and substrate mycelia, basitonously branched, 230-1020 um long, tapering from 6-10 um wide at the base to 2-4(-5) um at the tip; sporulation good on PCA, SEA and MA.	Sporangia: globose, many-spored, 20-30 um diam; wall dehiscence with minute or inconspicuous collarettes and a trace of a columella.	Chlamydospores: abundant, mostly terminalary, globose, (12-)20-41 um diam.	Sporangiospores: cylindrical, ellipsoidal, smooth-wallled, (4-)7-10(-16) × (4-)8-14(-20) um.	[44]
M. globalpina	Colony densely lobate on MA, aerial mycelia moderately abundant, white, cottony; garlic-like odour strong; colony 30 mm diam after 5 d at 20°C.	Sporangiophores arising from aerial mycelia, unbranched, (30-60-130(-160) um long, tapering from 3-6 um wide at the base to 1-3 um at the tip, slightly swollen at the base: sporulation good on PCA, no sporulation on MA.	Sporangia globose, many-spored, 8-18 um diam; wall dehiscence with conspicuous collarettes, columellae mostly inconspicuous.	Chlamydospores absent.	Sporangiospores globose or subglobose, smooth-walled, 2.0-3.5(-5.0) um diam.	[44]
M. horticola	Colony (on MA): densely lobate, white, with abundant aerial mycelia and strong garlic-like odour; 80 mm after 5 d at 20°C.	Sporangiophores: arising from aerial/substrate mycelia, unbranched, 45–200 μm long; tapering from 2.5–6 μm (base) to 0.5–1 μm (tip).	Sporangia: globose, single-spored; spores verrucose/echinulate, 8–12 μm diam (6–14 μm range); wall dehiscence with inconspicuou	Chlamydospores absent	Not described	[44]

			s collarettes, columella absent.			
M. humilis	Colony (on MA): lobate/densely lobate, white, moderately abundant cottony aerial mycelia, strong garlic-like odour; 50–70 mm after 5 d at 20°C.	Sporangiophores: mostly from aerial mycelia, mostly basitonously branched, 50–120 µm (20–240 µm range); tapering from 2.5–4 µm (base, 2.0–6 µm range) to 0.5–1 µm (tip, 0.5–1.5 µm range).	Sporangia: globose, single-spored, 4–12 µm diam (4–15 µm range), spiny/verrucose; wall dehiscence with minute/inconspicuous collarettes, columella absent.	Absent	Not described	[44]
M. indohii	Colonies lobate or zonate, aerial mycelia white, rather abundant, powder-like; garlic-like odour strong; colony 45-50 mm after 5 d at 20°C.	Not described	Sporangia absent.	Not described	Not described	[44]
M. minutissima	Colonies lobate or flowery, aerial mycelia white, moderately abundant, cottony; garlic-like odour moderate; colonies 25-50 mm after 5 d at 20°C.	Sporangiophores arising from aerial and substrate mycelia, unbranched or rarely branched, 60-140(-200) um long, tapering from (2-3-6 um wide at the base to (0.5-1-3(-4) um; sporulation good on SEA, moderate on PCA and no sporulation on MA.	Sporangia globose, 10-24 um diam, containing usually less than 20 spores; wall dehiscence with minute or inconspicuous collarettes, and without a trace of a columella.	Absent	Sporangiospores globose, 3.5-7.0 um diam, slightly verrucose.	[44]

M. multispora	PDA: 28°C 28d, 90mm, cottony, circular, with entire edge, velvety, flossy.	Sporangiophores 10–20 µm high (up to 50 µm) × 2–10 µm diam., erect, developed from aerial hyphae and broad at the tip (tip swollen, 5–10 µm wide), hyaline, smooth-walled, non-septate with or without branched, with granules.	Sporangia 20–50 × 20–50 µm ($\bar{x}$ = 37.5 × 39 µm, n = 20), globose to subglobose, 1-celled, unicellular, with multi-spores, hyaline, smooth and thick-walled.	Absent	Sporangiospores 2–12 × 2.5–12 µm ($\bar{x}$ =6× 5.58 µm, n = 40), globose to subglobose, hyaline, smooth-walled.	[48]
M. mutabilis	Colonies zonately lobate on MA, aerial mycelia white, abundant and cottony: garlic-like odour strong; colonies 6 mm after 5 d at 20°C.	Sporangiophores: mostly from aerial mycelia, acrotonously branched; main sporangiophores 150–500 µm long (100–670 µm range), 6–15 µm wide at base (4–20 µm range), 2–4 µm at tip (2–7 µm range); side branches 30–120 µm long (30–220 µm range); sporulation good on PCA/SEA, poor on MEA.	Sporangia: globose, many-spored, (14-22-35(-41) um diam; wall dehiscence with a conspicuously collarette and a minute columella.	Chlamydospores: sparse, lemon-shaped/subglobose, 6–16 µm diam (6–24 µm range).	Sporangiospores: globose, subglobose or ellipsoidal, (3.5-4.0-9.0(-17) um diam.	[44]
M. oedema	PDA:16°C 5d, 45mm, garlic smell, sparse aerial mycelia, with characteristic rosette pattern.	Absent	Oval or spherical, smooth, deliquescent, hyaline, 30.7–42.5 µm in diameter.	Absent	Oval or round, 2.2–3.1 µm long, 2.0–2.9 µm wide.	This study
M. parvispora	Aerial mycelia (on MA): sparse, white; weak garlic-like odour;	Sporangiophores: from aerial/substrate mycelia, abundantly branched (mostly	Sporangia: globose, light brown at maturity, 12–35 µm diam (12–20–35	Chlamydospores: sparse, ellipsoidal or globose, 6-8 x 8-16 um.	Sporangiospores: globose and subglobose, 2-3 um diam.	[44]

	colony 40 mm after 5 d at 20°C.	mesotonous), 110–410 μm long, tapering from 6–12 μm (base) to 2–5 μm (tip, 1–6 μm range); sporulation good on SEA/PCA.	μm range), many-spored; wall dehiscence with conspicuous collarettes, mostly no columella trace.			
M. polycephala	Colony cottony, aerial mycelium abundant, white; garlic like odour moderate; colony 50 mm diam after 5 d at 20°C.	Sporangiophores: arising from aerial mycelium, racemosely branched; main stem 270–360 μm long, tapering from 14.6–23.9 μm (base) to 3.2–5.3 μm (tip); side branches 13–40 μm long, 2.7–4.0 μm wide.	Sporangia: globose, 19–32 μm diam; wall dehiscence with no/minute collarettes, usually no columella trace.	Absent	Sporangiospores globose or subglobose, (5.3-8.0-14.6 um diam.	[44]
M. tibetensis	PDA:16℃ 7d, 59mm, garlic smell and a wet dog smell, with sparse aerial mycelia.	Unbranched, 112–406 μm in height, tapering from 4.2–6.9 μm to at the base to 1.6–2.6 μm at the apex.	Almost spherical in shape, smooth, deliquescent, 12.8–30.3 μm in diameter.	Absent	Mostly pentagonal or hexagonal, smooth, 1.8–5.9 μm long.	This study
M. triangularis	Colony: very slow growth at 0–16 ℃ on PDA; produces white cottony aerial mycelium on PDA; thin hyaline substrate mycelium with reduced aerial mycelium on LCA and	Sporangiophores: arising from aerial/substrate mycelium, triangular-shaped, sometimes with rhizoids; 47–112 μm tall (n=25); tapering from swollen base (4 μm) to tip (1.5–1.7 μm).	Sporangia: smooth, hyaline, often triangular (young), sometimes round; 11.5–19.5 × 9.5–19 μm (n=30); dehiscence with collarette.	Chlamydospores not observed.	Sporangiospores: hyaline, smooth-walled, cylindrical, 2.6 – 4.1 μm × 1.7 – 2.6 μm (n = 30) (Mean ± SE = 2.9 ± 0.8μm) subglobose in a few strains.	[18]

	WA; characteristic rosette pattern on PDA and LCA.					
M. turficola	Colony on 2% MEA (pH 7): moderately fast-growing, reaching 6–6.5 cm diam. in 6 days; scanty aerial mycelium mostly present; weak genus-typical odour	Sporangiophores: numerous, mostly from aerial hyphae, generally unbranched (rarely 1 basitonous lateral branch); 100–250 µm tall; 6–10 µm wide at base (6–17 µm range), tapering to 2–4 µm at tip.	Sporangia: mostly 20–32 µm diam, many-spored; dehiscence leaves prominent columella (2–5 µm high).	Absent	Sporangiophores: regularly globose, smooth-walled, 2.5-3.0(-4-5) µm in diameter.	[52]
M. verrucosa	Colony: (on MA): zonately lobate, white, abundant cottony aerial mycelia, strong garlic-like odour; 50–70 mm after 5 d at 20°C.	Sporangiophores: mostly from aerial mycelia, mostly basitonously branched, 70–250 µm long (70–330 µm range); tapering from 3–7 µm (base, 2–11 µm range) to 1–2 µm (tip, 0.5–3 µm range); sporulation good on MA, PCA, SEA.	Sporangia: globose, 10–28 µm diam (10–16–28 µm range), containing <20 spores; wall dehiscence with minute collarettes, mostly with inconspicuous columella.	Chlamydospores: abundant, ellipsoidal, 6–12 × 12–20 µm (6–18 × 12–26 µm range).	Sporangiospores: ellipsoidal, 6–12 µm diam (3–16 µm range), verrucose.	[44]
M. verticillata	Colony：(on MA): Pale Greyish Vinaceous to Russet-Vinaceous, velvety, with obvious zonate growth; 40 mm after 5 d at 20°C; garlic-like	Sporangiophores ： arising from substrate mycelia, septate, branched; 120–300 µm long (120–500 µm range); 4–8 µm wide at base (2–11 µm range), 2–4 µm at tip (1–4 µm range).	Sporangia ： globose, red-brown, many-spored, 10–20 µm diam; wall dehiscence with minute collarette; columellae minute/incon	Chlamydo-spores: ellipsoidal and subglobose, relatively abundant, 20-35 um diam.	Sporangiospores ： irregularly globose or subglobose, (2.0-)2.5-3.5(-4.5) um.	[44]

	odour absent.		spicuous, <3 μm high.			
M. wolfii	Colony：fast-growing, white/greyish-white, downy; surface broadly zonate/lobed (rosette-like); no reverse pigment.	Sporangiophores：typically erect, delicate, 80–250 μm tall, 6–20 μm wide at base; arising from rhizoids/bulbous swellings on substrate hyphae; terminating with compact cluster of short acrotonous (terminal) branches.	Sporangia：15–48 μm diam; walls transparent; conspicuous collarette usually present after sporangiospore dehiscence.	Chlamydospores with or without blunt appendages (amoeba-like) may be present	sporangiospores are single-celled, short-cylindrical, 6-10 x 3-5 μm, with a double membrane.	[54]
M. wuyishanensis	Colony：irregularly lobate, white, with rather sparse aerial mycelia and moderate garlic-like odour; reaching 80 mm after 5 days at 20°C.	Sporangiophores：mostly from aerial mycelia; highly variable in length, width, and branching; 40–330 μm long (10–580 μm range); tapering from 4–18 μm (base, 2–26 μm range) to 0.5–6 μm (tip, 0.5–9 μm range).	Sporangia：mostly irregular, frequently globose/subglobose, 8–43 μm diam, brown, many-spored; wall dehiscence with minute/inconspicuous collarettes, mostly columella absent.	Absent	Sporangiospores globose or subglobose, (3-4-6.5 um diam.	[44]
M. yunnanensis	PDA:28°C 28d, 90mm, spreading, with sparse aerial mycelium, irregular, with undulate edge, velvety, flossy.	Not found	Not found	Not found	Not found	[48]
M. zychae	PDA: (28℃,5d)colony 76–79	Sporangiophores: hyaline, erect, simple; 190–1,000	Sporangia: spherical, 15–35 μm	Chlamydospores: globose, catenulate,	Sporangiospores: hyaline, ellipsoidal,	[53]

	mm; front whitish, often dark whitish at center. MEA: (28℃,5d)colony 77–80 mm; pale whitish, broadly zonate with concentric pattern.	µm long, tapering toward apex; 8–12 µm wide at base, 2–4 µm at apex; bearing terminal sporangia.	diam, wrinkled, non-columellate.	clustered, 15–19 µm diam.	single-celled, 6–10 × 4–6 µm.	
Linnemannia rotunda	Colonies on PDA at 16°C for 5 d, reaching 88 mm diameter, garlic smell, with sparse aerial mycelia.	Sporangiophores erect or slightly bent, unbranched, 25.5–146.0 µm long, 1.9–5.0 µm wide	Sporangia oval to round, smooth, multi-spored, 10.4–22.3 µm long, 10.7–22.6 µm wide.	Chlamydospores present, mostly oval, 10.1–22.0 µm long, 6.6–15.6 µm wide.	Sporangiospores smooth, hyaline, mostly round, 9.6–19.0 µm in diameter.	This study
L. bainierella	Colony: fast-growing at 16°C on PDA, producing white cottony aerial mycelium; extremely translucent with poor aerial mycelium on LCA, SE, and WA; sporulation on PDA, LCA, WA; faint garlic odour.	Sporangiophores: arising from aerial and substrate mycelium, mesotonously branched, 350–600(–730) µm tall (n = 8), tapering from 6.6–10.1(–14) µm to 3.0–3.6 µm at the tip.	Sporangia hyaline, round, smooth-walled, 19–33 µm diam (n = 5), multi-spored, after spore liberation with distinct collarette, no columella.	Chlamydospores not observed.	Sporangiospores hyaline, cylindrical, smooth 5.0–7.0 × 2.5–4.0 µm (n = 30) (Mean ± SE = 4.9 ± 1.37 µm).	[18]
L. friederikiana	Colonies fast growing, no pattern formed, with	Sporangiophores: arising from aerial/substrate mycelium, with	Sporangia hyaline, round, smooth-	Chlamydospores thick-walled, appearing in pairs or cluster in the	Sporangiospores hyaline, smooth-walled and spiny, irregular	[18]

	white cottony aerial mycelium on PDA, extremely translucent on LCA, SE, and WA, sporulation on PDA, LCA, and SE, odour unpleasant of wet dog.	branched/unbranched types; unbranched 200–515 µm tall; pronounced branching on WA; mesotonously branched 292–700 µm tall (n=30), tapering from 9.5 µm (base) to 2.8–5.0 µm (tip).	walled, 18–37 µm (n = 30) after spore liberation with collarette and columella.	substrate mycelium on PDA and LCA.	in shape, spores mainly produced on the substrate mycelium, roundish, 9.2–18.2 × 8.9 16.8 µm (Mean ± SE = 12.2 ± 2.6 µm) (n = 30).	
L. mannui	Colonies fast-growing on PDA at 16°C, white cottony mycelium with white exudate droplets, no pattern, poor sporulation on PDA but good on WA and LCA. Odour: garlic and fruity.	Sporangiophores: On aerial and substrate mycelium, with rhizoids, unbranched or mesotoneous/acrotonous branching, 162–448 µm tall, tapering from 4.8–7 µm wide base to 1.4–1.5 µm at tip.	Sporangia: Hyaline, round, smooth-walled, 21–27 µm diameter; with inconspicuous collarette and columella after spore release.	Absent	Sporangiospores hyaline, smooth-walled, cylindrical, 5.5–11.5 × 4.0–11.0 µm (n = 30) (Mean ± SE = 7.3 ± 1.8 µm).	[18]
L. nimbosa	Colonies fast-growing on PDA, white cottony mycelium, no pattern. Sporulation on SE media. Strong garlic odour.	Sporangiophores: On aerial mycelium, occasional rhizoids on substrate, unbranched or acrotonous, 100–391 µm tall, tapering from 4.2–6.9 µm to 1.6–2.6 µm at tip.	Sporangia: Hyaline, round, smooth-walled, 12–31 µm diameter, few spores, with inconspicuous collarette and columella.	Intercalary and terminal chlamydospores present in substrate mycelium.	Sporangiospores: hyaline, smooth walled, cylindrical, often also irregular, 7.0–11.5 × 3.5–6.5 µm (n = 30) (Mean ± SE = 6.95 ± 2.13 µm).	[18]
L. scordiella	Colonies: Fast-growing, white cottony on	Sporangiophores: Long, unbranched, 160–300 µm tall, tapering from	Sporangia hyaline, smooth, round, multi-	Chlamydospores terminal and intercalary, thick-walled, smooth, 15–30 µm (n = 3).	Sporangiospores hyaline, smooth-walled, ellipsoidal, 6.0–10 × 4.0–5.5 µm	[18]

	PDA; translucent with poor mycelium on LCA, SE, WA. Sporulation on PDA, LCA, WA. Odour: strong garlic.	6.4–7.7 µm at base to 1.8–2.6 µm at tip, with columella and collarette after spore release.	spored 16–33 µm (n = 3).		(n = 30) (Mean ± SE = 6.07 ± 1.9 µm).	
L. stellaris	Colonies on PDA: fast-growing, white, cottony, rosette pattern, no sporulation. On LCA: star-like, poor sporulation. Odour: musty, garlic, crushed leaves.	Sporangiophores: 123–264 µm tall (avg. 5), unbranched or with mesotoneous branches, tip tapers from 4–5 µm to 3 µm, base not enlarged.	Sporangioles: hyaline, round, smooth, 11.5–28 µm diameter, few or single spores, with collarette and columella after spore release.	Chlamydospores terminal or intercalary, within the medium or on the surface, roundish, thick walled, sometimes with oil droplets 11–15 µm diam.	Sporangiospores hyaline and of two types, ovoid to round, smooth or warty, 8.5–15.5 × 7.5–19.0 µm (n = 30) (Mean ± SE = 10.65 ± 2.1 µm).	[18]
