# Supplementary material for: Unveiling Species Diversity Within Early-Diverging Fungi from China VIII: Four New Species in Mortierellaceae (Mortierellomycota)
Source: Microorganisms. 2025 Jun 7;13(6):1330. doi: 10.3390/microorganisms13061330 (PMC12195542; doi:10.3390/microorganisms13061330)
Supplement: Supplementary file 1 [file microorganisms-13-01330-s001.zip › Mortierellaeace supplementary materials.pdf]

Table S1. GenBank accession numbers of sequences used in this study.

Species	Strains	ITS	LSU	SSU	*RPB1*	*Act*
Linnemannia acrotona	CBS 386.71*	NR_111574	NA	NA	NA	NA
L. amoeboidea	CBS 889.72*	NR_111579	NA	NA	NA	NA
L. bainierella	Pr1s21	MT380864	MZ981756	NA	NA	NA
L. bainierella	Pr1s13	MT380862	NA	NA	NA	NA
L. bainierella	Pr1s20	MT380866	NA	NA	NA	NA
L. biramosa	SYFGD6-2	KP744414	NA	NA	NA	NA
L. biramosa	SYFGP2-1	KP744415	NA	NA	NA	NA
L. biramosa	RS5	PP703041	NA	NA	NA	NA
L. camargensis	CBS 221.58*	NR_111577	NA	NA	NA	NA
L. camargensis	CBS 221.58*	MH857763	MH869294	NA	NA	NA
L. elizabethkennyiae	BRIP 74948a*	NR_189983	OR259051	OR271910	NA	NA
L. elongata	7	PQ678932	NA	NA	NA	NA
L. exigua	NNIBRFG5521	ON715840	ON715849	NA	NA	NA
L. fatshederae	CBS 388.71*	NR_182454	MH871946	NA	NA	NA
L. fluviae	EML-YR25716-1*	KX227755	NA	NA	NA	NA
L. friederikiana	Pr3s8*	MT308723	MZ981755	NA	ON774872	NA
L. gamsii	CBS 749.68*	NR_152954	MH870946	MH859222	NA	NA
L. hyalina	CBS 223.35*	NR_163542	MH867166	MH855655	NA	NA
L. longigemmata	CBS 653.93*	NR_182440	NA	NA	NA	NA
L. mannui	Pr2s5	MW042230	MZ981765	NA	ON774871	NA
L. nantahalensis	CBS 610.70*	NR_145300	NA	NA	NA	NA
L. nimbosa	HFSF57*	MW042228	MZ981762	NA	ON774865	NA
L. rotunda	**CGMCC3.28764***	**PV113446**	**PV113454**	**PV113462**	**PV294740**	**PV278672**
L. rotunda	**XG08755-7-2**	**PV113447**	**PV113455**	**PV113463**	**PV294741**	**PV278673**
L. schmuckeri	CBS 295.59*	NR_111578	MH869405	MH857867	NA	NA
L. sclerotiella	CBS 529.68*	NR_145298	NA	NA	NA	NA
L. scordiella	HFSF81*	MW042238	MZ981760	NA	MZ779209	NA
L. solitaria	OAS3*	MT279272	NA	NA	NA	NA
L. stellaris	Ks2-4*	MW042232	MZ981764	NA	ON774866	NA
L. zychae	CBS 316.52*	NR_111576	NA	MH857054.1	NA	NA
Mortierella cogitans	CBS 879.97*	HQ630281	NA	NA	NA	NA

Table S2. GenBank accession numbers of sequences used in this study.

Species	Strains	ITS	LSU	SSU	*RPB1*	*Act*
Mortierella acrotona	CBS 386.71*	JX975921	HQ667405	HQ667489	NA	NA
M. acuta	**CGMCC3.28761***	**PV113442**	**PV113450**	**PV113458**	**PV294736**	**PV268287**
M. acuta	**XG08182-4-2**	**PV113443**	**PV113451**	**PV113459**	**PV294737**	**PV268288**
M. alpina	CBS 210.32*	JX975853	MH866743	JQ040258	JN985287	NA

M. alpina	CBS 210.32	HQ630345	HQ667421	HQ667501	NA	NA
M. amoeboidea	CBS 889.72*	HQ630346	HQ667422	HQ667502	NA	NA
M. angusta	CBS 293.61*	HQ630279	HQ667358	HQ667443	NA	NA
M. antarctica	CBS 609.70*	HQ630347	HQ667423	HQ667503	MN743904	NA
M. armillariicola	CBS 914.73*	HQ630282	HQ667361	HQ667446	NA	NA
M. bainieri	CBS 220.35	MH855653	MH867164	NA	NA	NA
M.basiparvispora	CBS 517.72*	JX976048	MH872255	MH860551	NA	NA
M. beljakovae	CBS 123.72*	HQ630352	HQ667428	NA	NA	NA
M. biramosa	CBS 370.95	JX976094	HQ667389	HQ667473	NA	NA
M. calciphila	WA 18944*	KT964845	NA	NA	NA	NA
M. camargensis	CBS 221.58*	HQ630331	HQ667408	HQ667492	NA	NA
M. capitata	CBS 110.640	JX975923	NA	NA	NA	NA
M. chlamydospora	CBS 120.34	HQ630354	HQ667430	HQ667508	NA	NA
M. clonocystis	CBS 357.76*	HQ630318	HQ667395	HQ667479	NA	NA
M. cogitans	CBS 879.97*	HQ630281	HQ667360	HQ667445	NA	NA
M. cystojenkinii	CBS 456.71*	HQ630348	HQ667424	HQ667504	NA	NA
M. dichotoma	CBS 221.35*	HQ630316	HQ667393	HQ667477	NA	NA
M. echinosphaera	CBS 575.75*	GU559985	HQ667431	NA	NA	NA
M. elongata	FSU823	HQ630337	HQ667413	HQ667495	NA	NA
M. elongata	FSU822	HQ630336	HQ667412	HQ667494	NA	NA
M. elongatula	CBS 488.70*	HQ630349	HQ667425	HQ667505	NA	NA
M. epicladia	CBS 355.76*	HQ630319	HQ667396	HQ667480	NA	NA
M. epigama	CBS 489.70*	HQ630290	HQ667367	HQ667453	NA	NA
M. exigua	CBS 655.68*	HQ630329	HQ667406	HQ667490	NA	NA
M. fimbricystis	CBS 943.70*	GU559986	NA	NA	NA	NA
M. formicicola	CBS 109.589	JX975933	JX976140	NA	NA	NA
M. formicae	WA 49853*	NR_160334	KY748017	KY748015	NA	NA
M. gamsii	CBS 749.68*	HQ630340	HQ667416	NA	NA	NA
M. gamsii	CBS 551.73	HQ630341	HQ667417	HQ667498	NA	NA
M. gemmifera	CBS 134.45*	HQ630293	HQ667371	HQ667456	NA	NA
M. globalpina	CBS 360.70*	NR_160121	MH871462	MH859709	NA	NA
M.histoplasmatoides	CBS 321.78*	HQ630309	HQ667386	HQ667470	NA	NA
M. horticola	CBS 305.52*	HQ630322	HQ667399	HQ667483	NA	NA
M. hypsicladia	CBS 116.202*	HQ630302	HQ667379	NA	NA	NA
M. humilis	CBS 222.35*	HQ630325	HQ667401	HQ667485	NA	NA
M. humilis	FSU828	HQ630326	HQ667402	HQ667486	NA	NA
M. humilis	CBS 745.68	HQ630327	HQ667403	HQ667487	NA	NA
M. indohii	CBS 720.71*	HQ630298	HQ667377	HQ667461	NA	NA
M. indohii	FSU830	HQ630299	EU736318	EU736291	NA	NA
M. indohii	FSU831	HQ630300	HQ667438	HQ667462	NA	NA
M. kuhlmanii	CBS 157.71*	HQ630294	HQ667372	HQ667457	NA	NA
M. lapis	OBS3*	MT380877	MZ981747	NA	ON774869	NA
M. lignicola	CBS 207.37*	HQ630357	HQ667435	HQ667511	NA	NA

M. longicollis	CBS 209.32*	HQ630287	HQ667365	HQ667451	JN985283	LN847392
M. macrocystis	CBS 314.85	JX975974	NA	NA	NA	NA
M. microzygospora	CBS 880.97*	HQ630317	HQ667394	HQ667478	NA	NA
M. minutissima	CBS 307.52	HQ630323	HQ667400	HQ667484	NA	NA
M. multispora	KUMCC 200005*	MT031921	MT032146	NA	NA	NA
M. mutabilis	CBS 308.52*	HQ630315	HQ667392	HQ667476	NA	NA
M. nantahalensis	CBS 610.70*	HQ630311	HQ667388	HQ667472	NA	NA
M. oedema	**CGMCC3.28762***	**PV113444**	**PV113452**	**PV113460**	**PV294738**	**PV278677**
M. oedema	**XG00420-1-2**	**PV113445**	**PV113453**	**PV113461**	**PV294739**	**PV278678**
M. paraensis	CBS 547.89	HQ630353	HQ667429	NA	NA	NA
M. parazychae	CBS 868.71*	HQ630283	HQ667362	HQ667447	NA	NA
M. parvispora	CBS 311.52	EU484279	HQ667373	HQ667458	NA	NA
M. polycephala	FSU696	HQ630332	HQ667409	HQ667493	NA	NA
M. polycephala	FSU866	HQ630333	HQ667410	NA	NA	NA
M. polygonia	CBS 685.71*	HQ630301	HQ667378	HQ667463	NA	NA
M. pulchella	CBS 312.52	HQ630351	HQ667427	HQ667507	NA	NA
M. rishikesha	CBS 652.68*	HQ630308	HQ667385	HQ667469	NA	NA
M. rostafinskii	CBS 522.70	HQ630358	HQ667436	HQ667512	NA	NA
M. sarnyensis	CBS 122.72*	HQ630313	HQ667390	HQ667474	NA	NA
M. schmuckeri	CBS 295.59*	HQ630338	HQ667414	HQ667496	NA	NA
M. sclerotiella	CBS 529.68*	HQ630310	HQ667387	HQ667471	NA	NA
M. selenospora	CBS 811.68*	HQ630343	HQ667419	HQ667499	NA	NA
M. simplex	CBS 243.82	JX975870	NA	NA	NA	NA
M. strangulata	CBS 455.67*	HQ630359	HQ667437	HQ667513	NA	NA
M. stylospora	CBS 211.32*	HQ630280	HQ667359	HQ667444	NA	NA
M. tibetensis	**CGMCC3.28763***	**PV113448**	**PV113456**	**PV113464**	**PV415185**	**PV278674**
M. tibetensis	**XG00421-2-2**	**PV113449**	**PV113457**	**PV113465**	**PV415186**	**PV278675**
M. triangularis	OAS8	MT380873	MZ981741	NA	ON774868	NA
M. turficola	CBS 432.76*	HQ630350	HQ667426	HQ667506	NA	NA
M. verrucosa	CBS 181.73	NA	MH878485	NA	NA	NA
M. verticillata	CBS 346.66	JN943799	JN940870	HQ667481	JN985284	NA
M. wolfii	CBS 209.69	HQ630303	HQ667380	HQ667464	JN985290	NA
M. wolfii	CBS 612.70	HQ630304	HQ667381	HQ667465	NA	NA
M. wolfii	CBS 651.93	HQ630305	HQ667382	HQ667466	JN985289	NA
M. wuyishanensis	CBS 370.95*	NR_152953	NA	NA	NA	NA
M. yunnanensis	KUMCC 200009*	MT031917	MT032142	NA	NA	NA
M. zonata	CBS 228.35*	HQ630356	HQ667433	NA	NA	NA
M. zychae	CBS 316.52*	HQ630330	HQ667407	HQ667491	NA	NA
Umbelopsis autotrophica	CBS 310.93*	HQ630285	HQ667363	HQ667449	NA	NA
U. isabellina	NRLL1757	HQ630284	NA	HQ667448	NA	NA

Notes: The newly discovered species identified in the present study are in bold. Ex-type strains are marked with a star marker "*". NA stands for “not available”.
